# Involvement of SUR2/Kir6.1 channel in the physiopathology of pulmonary arterial hypertension

**DOI:** 10.3389/fcvm.2022.1066047

**Published:** 2023-01-10

**Authors:** Hélène Le Ribeuz, Bastien Masson, Mary Dutheil, Angèle Boët, Antoine Beauvais, Jessica Sabourin, Vincent Thomas De Montpreville, Véronique Capuano, Olaf Mercier, Marc Humbert, David Montani, Fabrice Antigny

**Affiliations:** ^1^Université Paris-Saclay, Faculté de Médecine, Le Kremlin-Bicêtre, France; ^2^INSERM UMR_S 999 « Hypertension Pulmonaire Physiopathologie et Innovation Thérapeutique », Hôpital Marie Lannelongue, Le Plessis-Robinson, France; ^3^Hôptal Marie Lannelongue, Groupe Hospitalier Paris Saint-Joseph, Le Plessis Robinson, France; ^4^Inserm, UMR-S 1180, Signalisation et Physiopathologie Cardiovasculaire, Université Paris-Saclay, Orsay, France; ^5^Department of Pathology, Hôptal Marie Lannelongue, Groupe Hospitalier Paris Saint-Joseph, Le Plessis-Robinson, France; ^6^Service de Chirurgie Thoracique, Vasculaire et Transplantation Cardio-Pulmonaire, Hôpital Marie Lannelongue, Groupe Hospitalier Paris Saint Joseph, Le Plessis Robinson, France; ^7^Assistance Publique–Hôpitaux de Paris (AP-HP), Service de Pneumologie et Soins Intensifs Respiratoires, Centre de Référence de l’Hypertension Pulmonaire, Hôpital Bicêtre, Le Kremlin-Bicêtre, France

**Keywords:** ATP, ABCC9, pulmonary arterial tone, migration, proliferation, metabolism

## Abstract

**Aims:**

We hypothesized that the ATP-sensitive K^+^ channels (KATP) regulatory subunit (ABCC9) contributes to PAH pathogenesis. *ABCC9* gene encodes for two regulatory subunits of KATP channels: the SUR2A and SUR2B proteins. In the KATP channel, the SUR2 subunits are associated with the K^+^ channel Kir6.1. We investigated how the SUR2/Kir6.1 channel contributes to PAH pathogenesis and its potential as a therapeutic target in PAH.

**Methods and results:**

Using *in vitro, ex vivo*, and *in vivo* approaches, we analyzed the localization and expression of SUR2A, SUR2B, and Kir6.1 in the pulmonary vasculature of controls and patients with PAH as in experimental pulmonary hypertension (PH) rat models and its contribution to PAH physiopathology. Finally, we deciphered the consequences of *in vivo* activation of SUR2/Kir6.1 in the monocrotaline (MCT)-induced PH model. We found that SUR2A, SUR2B, and Kir6.1 were expressed in the lungs of controls and patients with PAH and MCT-induced PH rat models. Organ bath studies showed that SUR2 activation by pinacidil induced relaxation of pulmonary arterial in rats and humans. *In vitro* experiments on human pulmonary arterial smooth muscle cells and endothelial cells (hPASMCs and hPAECs) in controls and PAH patients showed decreased cell proliferation and migration after SUR2 activation. We demonstrated that SUR2 activation in rat right ventricular (RV) cardiomyocytes reduced RV action potential duration by patch-clamp. Chronic pinacidil administration in control rats increased heart rate without changes in hemodynamic parameters. Finally, *in vivo* pharmacological activation of SUR2 on MCT and Chronic-hypoxia (CH)-induced-PH rats showed improved PH.

**Conclusion:**

We showed that SUR2A, SUR2B, and Kir6.1 are presented in hPASMCs and hPAECs of controls and PAH patients. *In vivo* SUR2 activation reduced the MCT-induced and CH-induced PH phenotype, suggesting that SUR2 activation should be considered for treating PAH.

## 1. Introduction

Pulmonary arterial hypertension (PAH) is a severe and complex disease, defined as an elevation of >20 mmHg in the mean pulmonary artery (PA) pressure, pulmonary vascular resistance (PVR) of >2 Wood units at rest, and a PA wedge pressure of ≤15 mmHg (1). PAH is a complex and multifactorial disease that is associated with narrowing of the distal PA (diameter < 500 μm), leading to right ventricular (RV) hypertrophy and failure, and ultimately death (2). In the last two decades, more than 20 genes have been identified to be associated with a genetic predisposition to PAH, including two genes that encode for potassium channel proteins: *KCNK3* (Potassium Two Pore Domain Channel Subfamily K Member 3) and *ABCC8* (ATP-binding cassette subfamily C member 8) (3–5). In 2018, Bohnen et al. identified 12 loss of function (LOF) mutations in *ABCC8* (5). *ABCC8* encodes for the SUR1 protein, a regulatory subunit of ATP-sensitive-K^+^ channels (KATP). *ABCC8* mutation carriers are younger at diagnosis than those with idiopathic PAH (median age at diagnosis 14 years vs. 42 years) (5).

Four Kir6.x constitutes KATP channels (Kir6.1 or Kir6.2) and four sulfonylurea receptor subunits (SUR1 or SUR2A or SUR2B, depending on the cell type) (6). KATP channels are activated by reducing cytosolic ATP concentrations or elevation of nucleotide-diphosphate concentrations (7). Classically, SUR1/Kir6.2 channel is described to constitute the pancreatic KATP. However, we have previously shown that SUR1/Kir6.2 are expressed in pulmonary circulation and that SUR1 activation may be considered a therapeutic target for PAH (8). SUR2, encoded by the *ABCC9* gene, can be spliced into two isoforms: SUR2A or SUR2B. SUR2A/Kir6.2 channel is generally considered the cardiac KATP, while SUR2B/Kir6.2 and SUR2A/Kir6.1 are usually vascular KATP channels (7).

The pharmacological SUR1 activators used in the previous study could also weakly activate SUR2, as the transcripts for SUR2B and Kir6.1 were previously observed in hPASMCs and rat pulmonary arteries (9). Additionally, the selective SUR2 activator, pinacidil, induced pulmonary artery relaxation (10), and the non-selective KATP channel opener iptakalim inhibited the proliferation of control hPASMCs (11). Moreover, previous work suggested that global KATP channel opener nicorandil or iptakalim reduced the severity of PH in the preventive approach (12–14). We hypothesized that SUR2 and Kir6.1 could be additional actors in the pulmonary circulation and PAH pathogenesis. We investigated the localization, expression, and function of SUR2A, SUR2B, and Kir6.1 in controls, PAH-human pulmonary arterial endothelial cells (hPAECs), and human pulmonary arterial smooth muscle cells (hPASMCs), and experimental models of pulmonary hypertension (PH). We evaluated the consequences of SUR2/Kir6.1 channel activation in the proliferation rate and migration of hPASMCs and hPAECs. We assessed the role of the SUR2/Kir6.1 channel in pulmonary arterial tone using myograph experiments on isolated PA from control rats and the monocrotaline (MCT)-induced PH rat model (MCT-PH). Additionally, we assessed the effect of pharmacological activation of SUR2 with pinacidil on healthy rats and pre-clinical PH rat models (MCT and chronic-hypoxia rats) in curative protocol at 1 mg/kg/day.

## 2. Materials and methods

### 2.1. Patients

Human lung tissues were obtained at lung transplantation in 12 patients with PAH and upon pneumonectomy or lobectomy for restricted lung cancer from 10 control subjects. PAs were isolated away from tumor areas in the lung specimens of control subjects. Transthoracic echocardiography was performed pre-operatively in the control subjects to rule out PH (15). The demographic information of PAH and control patients are presented in Supplementary Table 1.

The patients studied were part of the French Network on Pulmonary Hypertension, a program approved by our institutional Ethics Committee, and had signed informed consent forms (Protocol N8CO-08- 003, ID RCB: 2008-A00485-50, approved on June 18, 2008). All human tissues were obtained with signed informed consent from transplant recipients or families of organ donors in accordance with the Declaration of Helsinki.

### 2.2. Human PASMC and PAEC culture

Pulmonary artery were excised away from tumor areas. hPAECs and hPASMCs were cultured as described previously (16, 17) and were used in passages 4–5 for the study. Patients studied were part of a program approved by the institutional Ethics Committee and had given written informed consent (ID RCB: 2018-A01252-53, approved on June 18, 2006).

### 2.3. Pulmonary vascular cell proliferation measurement

Cell proliferation was evaluated by quantifying the incorporation of BrdU using a DELFIA cell proliferation kit (AD0200, PerkinElmer) accordingly to the kit recommendations. Different treatments were used to evaluate the cells’ proliferation state: hPASMCs and hPAECs from control and PAH subjects were treated for 24 h with pinacidil (10 μmol/L) or the same amount of DMSO. Cell proliferation was stimulated by a medium containing 10 percent of FBS + EGF + insulin in the presence of 1 μmol/L BrdU for 24 h (Perkin Elmer). The fluorescence signal in the 96-well plate was read with an Envision 2103 plate reader (Perkin Elmer).

### 2.4. Animals and surgical procedures

The animal facility is licensed by the French Ministry of Agriculture (agreement N°C92-019-01). This study was approved by the Committee on the Ethics of Animal Experiments (CEEA26 CAP Sud). The animal experiments were performed in compliance with the guidelines from Directive 2010/63/EU on 22 September 2010 of the European Parliament on the protection of animals used for scientific purposes and complied with the French institution’s animal care and handling guidelines.

*In vivo* experiments performed in the study were performed according to clinical trial standards: animals were randomly dispersed between groups, and we performed blinded analyses. All rats received a number known by only the experimenter who administered the treatment to rats. Before euthanasia (day 21), all rats underwent an evaluation with closed-chest right heart catheterization. This experiment was performed by a blinded experimenter who did not know the correspondence between ID and treatment. Hemodynamic parameters were analyzed blindly.

Male Wistar rats (4 weeks old) were used in different experimental procedures:

1. PH was induced by a single MCT injection (60 mg/kg, s.c.). MCT was dissolved in 1 N HCl and neutralized with 1 N NaOH. Control animals received the same volume of saline solution.

2. Pinacidil-exposed rats: Wistar rats were treated with pinacidil (1 mg/kg/day in DMSO, daily intraperitoneal injection) from day 0 to day 21. Pinacidil was dissolved in DMSO. Control animals received the same volume of DMSO.

3. MCT-exposed Wistar rats were treated with pinacidil by intraperitoneal injection (1 mg/kg/day dissolved in DMSO) from days 14 to 21 and from days 0 to day 21 with a chronic protocol. Pinacidil was dissolved in DMSO. Control animals received the same volume of DMSO.

### 2.5. Anesthesia and euthanasia

Rats were placed under general anesthesia (induction: isoflurane 5% at room air) and spontaneous breathing with an isoflurane Rodent Anesthesia System (Minerve Esternay, France) (maintenance: isoflurane 2% at room air). At the end of experimental procedures, animals were euthanized under general anesthesia (isoflurane 5%) by cervical dislocation.

### 2.6. Chemicals

MCT and pinacidil were obtained from Sigma. U46619 was obtained from R&D Systems.

### 2.7. Western blot analyses

Total protein from human or rat lungs or isolated PA tissue samples were prepared as described previously (8). The list of antibodies used is presented in Supplementary Table 2.

### 2.8. Reverse transcription-quantitative PCR (RT-qPCR)

Total RNA was extracted using TRIzol. One μg of total RNA was reverse-transcribed using a QuantiTect Reverse Transcription Kit (Qiagen, Valencia, CA, USA; cat. no. 205311). Gene expression was quantified using qPCR following the standard protocol for ready-to-use TaqMan gene expression assays on a StepOne Plus Real-Time PCR System (Life Technologies). Pre-designed probe sets used for experiments are described in Supplementary Table 3.

### 2.9. Hemodynamic measurements and tissues collection

Under general anesthesia (isoflurane 2% at room air), hemodynamic measurements, such as right ventricular systolic pressure (RVSP; mmHg), cardiac output (CO; ml/min), and mean carotid pressure (mCP; mmHg), were measured blindly in unventilated anesthetized rats using a closed chest technique. Hemodynamic parameters were blindly analyzed.

After catheterization, animals were euthanized under general anesthesia (isoflurane 5%) by cervical dislocation. Then tissues were collected, and Fulton’s index (RV/LV + S) was calculated by weighing RV and LV plus septal (S).

### 2.10. Adult rat right ventricular myocytes isolation

Hearts from control rats were mounted on a Langendorff apparatus and perfused through the aorta with collagenase A (Roche, Meylan, France). The solution used to isolate myocytes was a Hanks-Hepes buffer solution containing (mM): NaCl, 117; KCl, 5.7; MgCl2, 1.7; KH2PO4, 1.5; NaHCO3, 4.4; HEPES, 21; glucose, 11.7; creatine, 10; taurine, 20; bubbled with 100% O2; pH 7-1. Digestion time varied between 40 and 50 min. After the enzymatic digestion, the right ventricles (RV) were excised, chopped finely, and agitated manually to dissociate individual myocytes.

### 2.11. Electrophysiological recordings

Borosilicate glass pipettes (Harvard Apparatus) were pulled with a Sutter puller, fired polished, and had a resistance between 1–2 MΩ. Series resistance was compensated up to 50% and was continually monitored during the experiment. The composition of the standard extracellular solution used to record APs was (mM): NaCl, 140; KCl, 4; CaCl2, 1.8; MgCl2, 1.1; HEPES, 10; glucose, 10; pH 7.4 (LiOH). When APs were recorded, the pipette solution contained (mM): KCl, 135; MgCl2, 4; Ethylene glycol-bis (2-aminoethyl ether)-N, N, N’, N’-tetraacetic acid (EGTA), 10; Glucose 10; HEPES, 10; Na2-ATP 5; Na2-CP 3, pH 7.2 (LiOH). In a current-clamp configuration, action potential was measured in response to brief depolarizing current (1–2 ms) injections at 1 Hz as described (18).

### 2.12. Myograph experiments

Human PAs, rat PAs, and descending aorta were mounted in an emkaBATH4 modular tissue bath system (EMKA Technologies, Paris, France) coupled to IOX software (EMKA Technologies). Human PAs were set at optimal length by equilibration against a passive load of 0.6 g. Rat PAs were set at 0.250 g and the aorta at 1 g.

Vessels were bathed in Krebs solution containing (in mmol/L) 119 NaCl, 4.7 KCl, 2.5 CaCl_2_, 1.17 MgSO_4_, 1.18 KH_2_PO_4_, 25 NaHCO_3_, and 11 glucose at 37°C and continuously aerated with a mixture of CO_2_/O_2_ (5%/95%). Rat PAs were set at optimal length by equilibration against a passive load of 0.3 g. After adding Krebs solution, vessels were contracted with 100 mmol/L K^+^-containing solutions (K100). Once a plateau was reached, the vessels were washed with Krebs solution for 30 min. Pinacidil (10 μmol/L) or the same volume of vehicle (DMSO) was used as pretreatments before the dose-response to KCl (10–90 mmol/L). After vessel contraction mediated by the thromboxane A2 analog U46619 (1 μmol/L), pinacidil was used to induce relaxation by increasing concentration (1 nmol/L to 100 μmol/L). Various KCl concentrations (K10 to K100) were prepared with an equimolar substitution of NaCl to maintain constant osmolarity and [Cl^–^] compared to standard Krebs solution. The contractile responses were normalized to the maximal response obtained with the K100 challenge. The relaxation responses were expressed as the percentage of the maximum contraction obtained with U46619.

### 2.13. Echocardiographic measurement

Trans-thoracic echocardiography (TTE) was performed with a digital ultrasound system (Vivid E9, GE Healthcare) using a high-frequency phased array transducer (12 S-D 4-12 MHz, GE Healthcare). The echocardiographic assessment was performed under general anesthesia and spontaneous breathing with an Isoflurane Rodent Anesthesia System (Minerve, Esternay, France) (maintenance isoflurane 2% at room air). Rats were shaved, and body temperature was controlled during experiments. Rats’ experimental conditions were unknown by the operator during TTE examination and data interpretation. Measurement of pulmonary artery acceleration time (PAAT), heart rate (HR), and pulmonary artery velocity time integral (VTI) were performed as previously described (18). In the 4-cavity view performed, we measured RV and LV thickness, RV or LV end-diastolic diameter (RV EDd, LV EDd, respectively), and systolic diameter (RV EDs, LV EDs). RV and LV fractional shortening (FS) correspond to the percentage change in LV and RV cavity diameters. LV FS (%) = (LV EDd-LV EDs/LV EDd) * 100 or RV FS (%) = (RV EDd -RV EDs/RV EDd) * 100.

### 2.14. Immunofluorescence staining

Paraffin-embedded thick sections of lung samples (5 μm thickness) were mounted on SuperFrostPlus slides (Thermo Scientific, Villebon sur Yvette, France). Slices were saturated with human serum (10%) in PBS for 1 h at room temperature. We used primary antibodies against SUR2A (1/100), SUR2B (1/100), or Kir6.1 (1/100) against α-Smooth Muscle Actin (α-SMA, Sigma, F3777) (1/200). Primary Antibody was detected with the secondary antibodies goat anti-mouse and goat anti-rabbit (1/400). Slides were counterstained with 4′,6′-diamidino-2-phénylindole (DAPI).

Pulmonary vessel neovascularization was evaluated by immunostaining against alpha-SMA-FITC (SMC marker F3777 Sigma)/Von Willebrand Factor (endothelial marker, A0082 DAKO). Immunostaining was quantified under an LSM 700 microscope (Carl Zeiss, Le Pecq, France). Images were recorded and analyzed with ZEN software (Carl Zeiss).

### 2.15. Wound healing assay

After 48 h of starvation (medium without growth factors: FBS, EGF, and insulin), human PASMCs were plated in a culture insert (Cat. No. 90209; Ibidi) at a density of 1.2 × 10^4^ cells per well in a fresh medium with cytosine arabinoside (10 μmol/L). After allowing cells to attach for 24 h, we removed the culture insert and washed the cells with phosphate-buffered saline to remove non-adherent cells. We added fresh medium with DMSO or pinacidil (at 10 μmol/L). We photographed the wound at time 0 and after 15 h for hPASMCs and between 0 and 8 h for hPAECs (corresponding to approximately 50% of wound recovery). Cell migration into the wound space was quantified using image J. Cell motility/invasion was assessed by the percentage of wound closure 15 h after initiation of wound healing {[(area T0–area T15)÷area T15] × 100}.

### 2.16. Pulmonary vessel and right ventricular remodeling analyses

The lungs were fixed in 4% paraformaldehyde, embedded in paraffin, and serially sectioned (5 μm). Pulmonary vascular remodeling was assessed in all the pulmonary vessels larger than 50 μm and less than 100 μm identified in 20 randomly selected microscopic fields per tissue section. The wall thickness was calculated according to the following equation: [External diameter–Internal diameter)/(External diameter)] × 100, as previously described (19).

Hearts were fixed in 4% paraformaldehyde, paraffin-embedded, and serially sectioned (5 μm). Heart sections were stained with Sirius red (0.1%) to assess heart fibrosis. As previously described, RV fibrosis was quantified using Image J software (20).

### 2.17. Statistical analyses

All statistical tests were performed using GraphPad Prism software (GraphPad, version 9.0 for Windows).

After checking with the Shapiro–Wilk, Kolmogorov-Smirnov, D’Agostino and Pearson test and Anderson-Darling Tests normality test whether the sample data followed a normal distribution, differences between the two were assessed using an unpaired *t*-test or Mann-Whitney test when conditions of parametric tests were not met. Kruskal-Wallis tests with *post-hoc* Dunn were used to compare three or more groups (all data with sample size *n* < 6/group and skewed data with sample size *n* ≥ 6/group) or one-way ANOVA with Dunnett *post-hoc* test for normally distributed data with sample size *n* ≥ 6/group. All values are reported as mean ± standard error of the mean. Representative images/figures were chosen to represent the mean of each quantification. For all experiments, *P-*value of <0.05 was considered statistically significant.

## 3. Results

### 3.1. The SUR2/Kir6.1 channel is expressed in the lungs of patients with PAH

**FIGURE 1:**
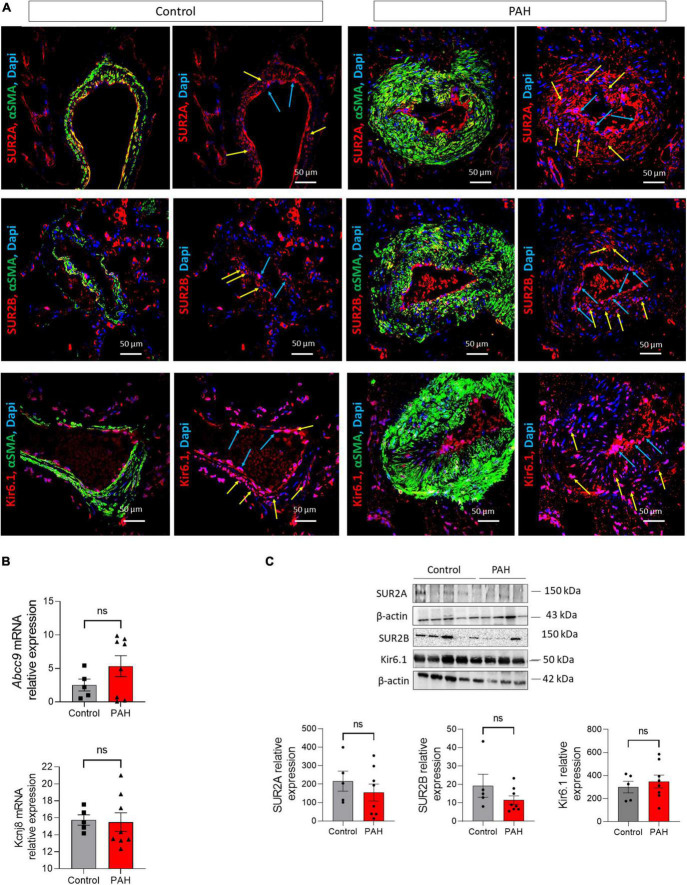
Expression of SUR2A, SUR2B, and Kir6.1 protein in lungs from control and patients with PAH. **(A)** SUR2A, SUR2B, or Kir6.1 immunostaining in paraffin-embedded lung sections from controls and patients with PAH; PAEC (blue arrow) and PASMC (yellow arrow) staining is visible. Scale Bar 50 μm. mRNA expression and representative western blots of *ABCC9* (coding for SUR2A and SUR2B) and *KCNJ8* (coding for Kir6.1) in the lungs of controls and patients with PAH [**(B,C)**, respectively] (*n* = 9–12). ns: non-significant vs. control. Two-tailed unpaired Student *t*-tests assessed the difference between the two groups.

To examine the localization and the expression of SUR2A, SUR2B, and Kir6.1 in controls and patients with PAH, paraffin-embedded lung sections of controls and patients with PAH were immunostained (Figure 1A). In both conditions, SUR2A, SUR2B, and Kir6.1 were expressed in hPAECs (blue arrow) and hPASMCs [yellow arrow, colocalization with smooth muscle actin isoform α (αSMA, in green)]. We subsequently quantified the relative amount of *ABCC9* and *KCNJ8* (coding for Kir6.1) mRNA levels in controls and patients with PAH, which showed that *ABCC9* and *KCNJ8* mRNA levels were unchanged in the lungs of PAH patients (Figure 1B). We performed western blot analysis using proteins from controls and PAH patients to determine the relative quantity of SUR2A, SUR2B, and Kir6.1 in the lung tissues. We found similar expression amounts of SUR2A, SUR2B, and Kir6.1 protein in the lung tissues of PAH patients and controls (Figure 1C).

### 3.2. Pharmacological activation of the SUR2/Kir6.1 channel reduced the proliferation of control-hPAECs

**FIGURE 2:**
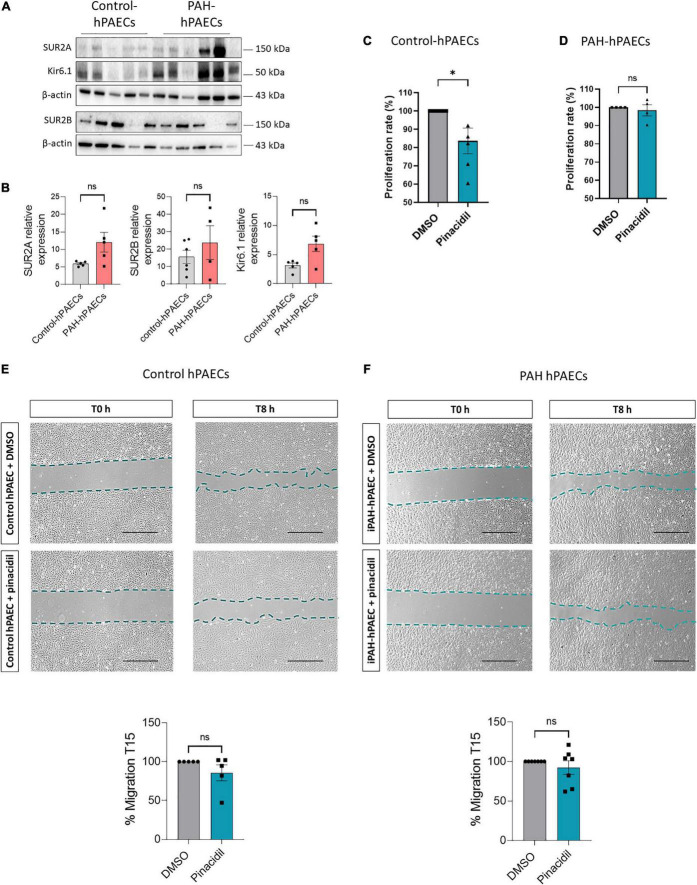
Pharmacological activation of SUR2/Kir6.1 channel reduced the proliferation in control hPAECs. **(A)** Representative western blots of SUR2A, SUR2B, and Kir6.1 in hPAECs in the lungs of controls and patients with PAH. **(B)** Quantification of SUR2A, SUR2B, and Kir6.1 expression in hPAECs from controls and patients with PAH (*n* = 4–6). **(C,D)** Analysis by Bromodeoxyuridine (BrdU) assay of the proliferation rate of hPAECs from controls and patients with PAH treated with pinacidil (10 μmol/L) or DMSO (*n* = 4–5 patients). **(E,F)** Analysis of the migratory capacity of hPAECs from controls and patients with PAH treated with pinacidil (10 μmol/L) or DMSO. Scale Bar = 500 μm (*n* = 5–7 patients). ns: non-significant. *p < *0.0*5. Two-tailed unpaired Student *t*-tests assessed the difference between the two groups.

Immunostaining revealed the expression of SUR2A, SUR2B, and Kir6.1 in the hPAECs. Using western blot analysis, we quantified their relative expression in the primary hPAECs. As shown in Figure 2A, SUR2A, SUR2B, and Kir6.1 protein were expressed in both control- and PAH-hPAECs. Compared to control-hPAECs, the expression of SUR2A and SUR2B proteins was unchanged, and Kir6.1 protein expression was increased in the PAH-hPAECs (Figure 2B). Therefore, we evaluated the role of SUR2 in the proliferation and migration of hPAECs following pharmacological activation of SUR2 with pinacidil (21, 22) (10 μmol/L) for 24 h. We chose to use pinacidil as an activator of SUR2 because contrary to other KATP channels, pinacidil activates SUR2B and SUR2A similarly (23) and has a very low affinity for SUR1 (24). Interestingly, pharmacological activation of SUR2 reduced the proliferation rate of control-hPAECs without altering the proliferation of PAH-hPAECs (Figures 2C, D). SUR2 activation by pinacidil had no consequence on the migration capacity of hPAECs in control- and PAH-hPAECs (Figures 2E, F).

### 3.3. Pharmacological activation of SUR2/Kir6.1 channel reduced the proliferation and migration of control- and PAH-hPASMCs and produced PA relaxation in PAH patients

**FIGURE 3:**
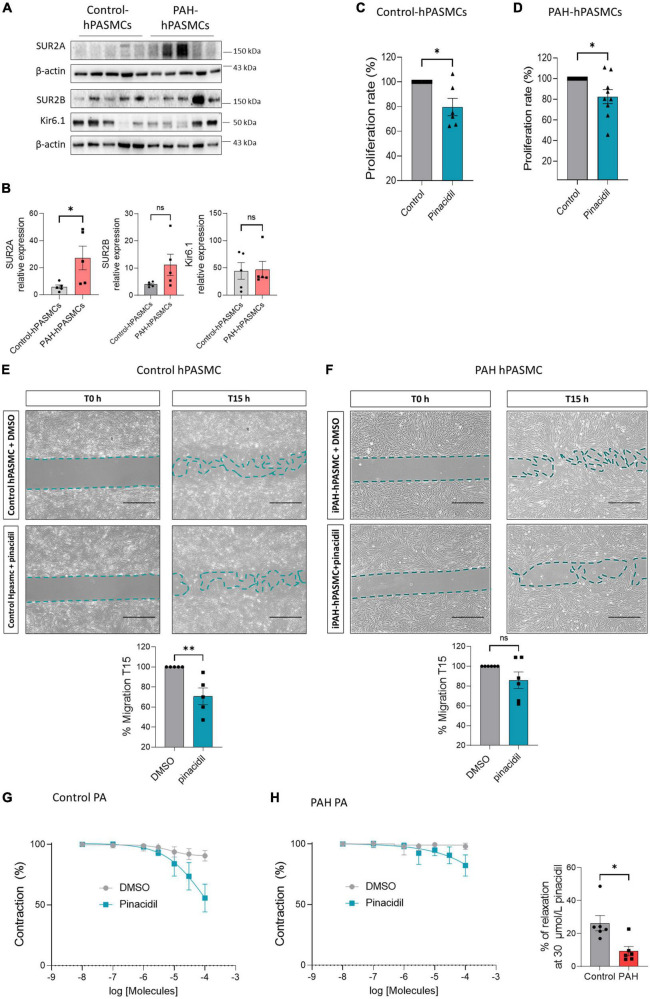
Pharmacological activation of SUR2/Kir6.1 channel reduced the proliferation and migration of control and PAH-hPASMCs. **(A)** Representative western blots **(A)** and quantification **(B)** of SUR2A, SUR2B, and Kir6.1 in hPASMCs from controls and patients with PAH (*n* = 5). Analysis by BrdU assay of the proliferation rate of hPASMCs from controls patients **(C)** and patients with PAH **(D)** treated with pinacidil (10 μmol/L) or DMSO (*n* = 6–7 patients). **(E,F)** Analysis of the migratory capacity of hPASMCs from controls **(E)** and patients with PAH **(F)** treated with pinacidil (10 μmol/L) or DMSO (*n* = 5–6 patients). Scale Bar = 500 μm. **(G)** Dose-response to pinacidil (100 nmol/L to 100 μmol/L) and DMSO on pre-contracted human PA control and iPAH **(H)** by 1 μmol/L of U46619. Graphics represent the relaxation percentage at 30 μmol/L of PA treated with pinacidil or DMSO (*n* = 6 patients). ns: non-significant. *p < *0.0*5 ^**^p < *0.001*.

We found that SUR2A, SUR2B, and Kir6.1 proteins were expressed in the control- and PAH-hPASMCs (Figure 3A). SUR2A protein was higher in the PAH-hPASMCs than in the controls. Further, SUR2B expression in the PAH-hPASMCs also tended to increase, though the increase was not significant (*p* = 0.053). Kir6.1 protein expression was unchanged in the PAH-hPASMCs (Figures 3A, B). Pharmacological activation of SUR2 reduced the proliferation rate of hPASMCs equally in both controls and PAH-hPASMCs (Figures 3C, D). SUR2 activation by pinacidil reduced the migration capacity of the control-hPASMCs but not the PAH-hPASMCs (Figures 3E–H).

We measured the consequence of SUR2 activation on the relaxation of PA in control and PAH patients (Figure 3G). We found pinacidil induced more potent PA relaxation in controls than in PAH conditions, suggesting that SUR2 contributes to the regulation of human PA tone, but SUR2-mediated PA relaxation was reduced in PAH patients.

### 3.4. SUR2 pharmacological activation produces the relaxation of PA in control and MCT-PH rats

**FIGURE 4:**
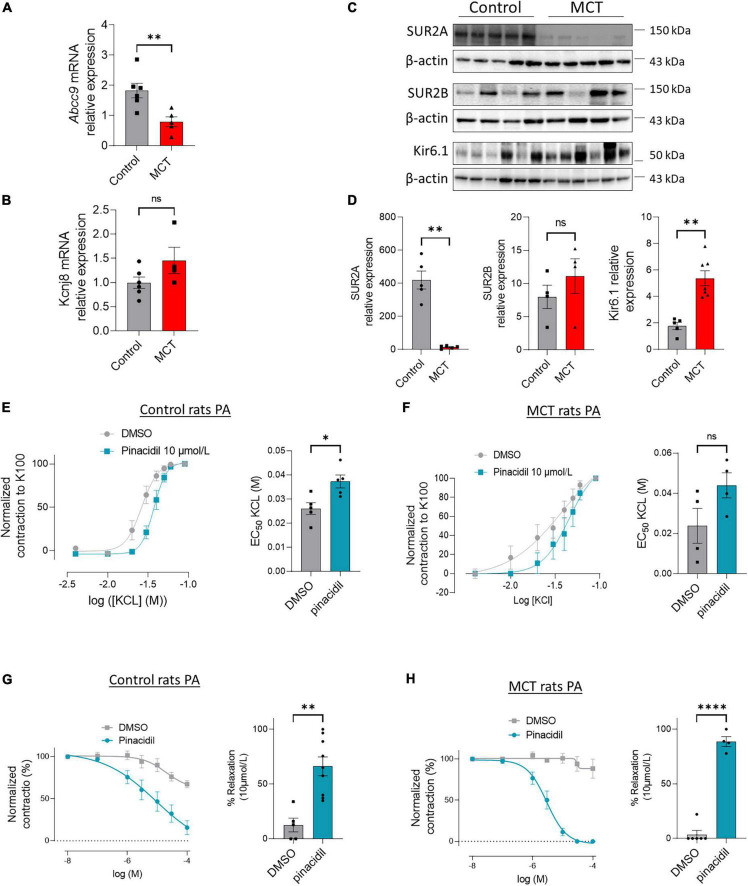
Pharmacological activation of SUR2 produces PA relaxation in control and MCT-PH rats. **(A,B)** *ABCC9* and *KCNJ8* mRNA expression was quantified in the lungs of control and MCT-PH rats (*n* = 4–6). **(C)** Representative western blots of SUR2A, SUR2B, and Kir6.1 in lungs from control and MCT-PH rats (3 weeks). **(D)** Quantification of SUR2A, SUR2B, and Kir6.1 expression in lungs of controls and MCT-PH rats (*n* = 4–6). **(E)** Dose-response curve (normalized to K100) was established by increasing concentrations of potassium chloride (KCl) to isolated PA from control rats in the presence of DMSO or pinacidil (SUR2 activator, at 10 μmol/L). Corresponding quantification EC50 values (*n* = 5 rats). **(F)** Dose-response curve (normalized to K100) was established by increasing concentrations of potassium chloride to isolated PA from control and MCT-PH rats in the presence of pinacidil at 10 μmol/L. Corresponding quantification EC50 values (*n* = 4 rats). **(G)** Dose-response to pinacidil (100 nmol/L to 100 μmol/L) and DMSO on precontracted control rat PA by 1 μmol/L of U46619. Graphics represent the contraction percentage at 10 μmol/L of PA treated with pinacidil or DMSO (*n* = 5–9 rats). **(H)** Dose-response to pinacidil (100 nmol/L to 100 μmol/L) and DMSO on pre-contracted MCT-PH rat PA by 1 μmol/L of U46619 (*n* = 4–5 rats). Graphics represent the contraction percentage at 10 μmol/L of PA treated with pinacidil or DMSO. ns: non-significant. *p < *0.0*5, ^**^p < *0.01*, ^****^p < *0.0001*.

We showed that the mRNA levels of *ABCC9* were reduced in the lungs of MCT-PH rats, while *KCNJ8* mRNA remained unchanged (Figures 4A, B). In addition, SUR2A expression was severely reduced, SUR2B expression was unchanged, and Kir6.1 expression increased in the MCT-PH rats’ lungs (Figures 4C, D).

Next, we investigated the consequence of SUR2 activation in the contraction and relaxation of PA from the controls and MCT-PH rats. In the controls, the contractile response to increasing concentration of potassium chloride (KCl, 10 to 90 mmol/L) was shifted significantly to the right in the presence of pinacidil (Figure 4E), as indicated by increased EC50 values (Figure 4E). In PA isolated from the MCT-PH rats, we found that pinacidil application induced similar results in PA from control rats (Figure 4F and Supplementary Figure 1).

After pre-contraction of PA with 1 μmol/L U46619, we applied increasing concentrations of pinacidil or dimethyl sulfoxide (DMSO) (100 nmol/L to 100 μmol/L). As shown in Figure 4G, in the controls, the contraction of PA was progressively reduced by increasing the dose of pinacidil (until almost total PA relaxation) compared to increasing the dose of DMSO, indicating that SUR2 activation-mediated relaxation of PA was showed by the decrease in PA contraction (Figure 4H). Similar results were obtained in the PA isolated from MCT-PH rats (Figure 4F and Supplementary Figure 1).

SUR2 activation induced more potent relaxation of PA in the MCT-PH rats than in the controls, suggesting that pinacidil-mediated PA relaxation mainly depends on SUR2B expression in MCT-PH rats. In contrast, SUR2A expression was impaired in the MCT-PH rats.

As presented in Supplementary Figure 2A, in the absence of endothelium, the relaxation mediated by pinacidil was diminished by 40% (Supplementary Figure 2A), suggesting that the endothelial-SUR2 also contributes to the regulation of pulmonary arterial tone.

### 3.5. Expression of SUR2A, SUR2B, and Kir6.1 in the hearts of MCT-PH rats

**FIGURE 5:**
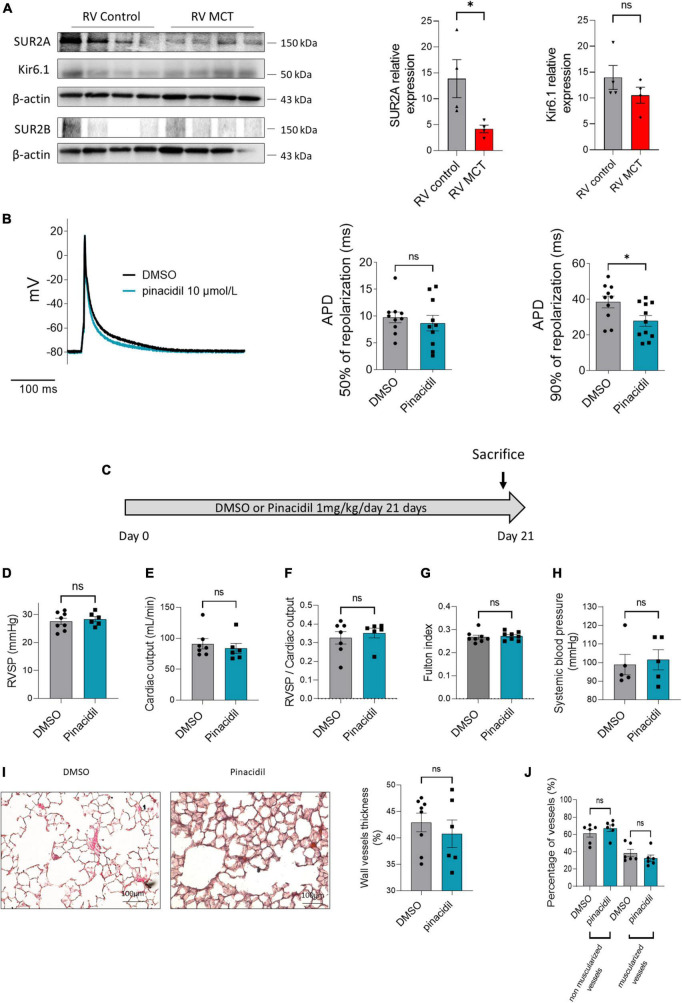
Consequences of SUR2 pharmacological activation in the RV compartment (*in vitro* and *in vivo*). **(A)** Representative Western blots of SUR2A, SUR2B1, and Kir6.1 in RV from control and MCT-PH rats (3 weeks), and quantification of SUR2A, SUR2B, and Kir6.1 expression in RV from control and MCT-PH rats (*n* = 5–8). **(B)** Representative action potential in RV cardiomyocytes isolated from control rats in DMSO or after perfusion of pinacidil. Analysis of action potential duration at 50 and 90% of AP depolarization in basal conditions and the presence of 10 μmol/L of pinacidil (12–14 cells from four different rats). **(C)** *In vivo* experimental design. Pinacidil (1 mg/kg^/^day for 3 weeks) was administered long-term to healthy control rats by intraperitoneal injection. **(D)** RVSP (mmHg; *n* = 6–8 rats per condition). **(E)** CO (mL/min) (*n* = 6–8 rats per condition). **(F)** PVR (*n* = 5 rats per condition) **(G)** Fulton index (*n* = 6–8 rats per condition) **(H)** and carotid artery mean pressure (*n* = X–X rats). **(I)** Pulmonary vessel occlusion (%) was analyzed by hematoxylin-eosin Safran staining (HES) (*n* = 6–8 rats per condition). **(J)** Percentage of non-muscularised and muscularised vessels [100 vessels per rat, (*n* = 6–8 rats)] as measured by immunostaining against α-smooth muscle actin (αSMA) and Von Willebrand Factor (VWF). ns: non-significant. *p < *0.05*.

Because SUR2 and Kir6.1 are expressed in the heart, we quantified the expression of SUR2 and Kir6.1 protein in the RV of control and MCT-PH rats by western blot analyses. In comparison with control rats, we found a reduced expression of SUR2A and an unchanged expression of Kir6.1 in the MCT-PH rats. SUR2B protein expression was not quantifiable in the RV of the control and MCT-PH rats (Figure 5A). At the same time, SUR2B appears to be expressed in the left ventricular (LV) (Supplementary Figure 3B). Moreover, SUR2A protein was decreased, and SUR2B and Kir6.1 expression were unchanged in the LV of the MCT-PH rats (Supplementary Figure 3B).

To study the contribution of SUR2 on RV action potential, we measured the effect of pinacidil on action potential duration (APD). The intake of pinacidil led to a shortening of APD without changes in resting membrane potential (Figure 5B, left panel). In the control RV cardiomyocytes, APD at 50% of AP repolarization was not affected by pharmacological activation of SUR2 (Figure 5B, middle panel). In contrast, APD at 90% of AP repolarization was significantly decreased (Figure 7B, right panel).

**FIGURE 6:**
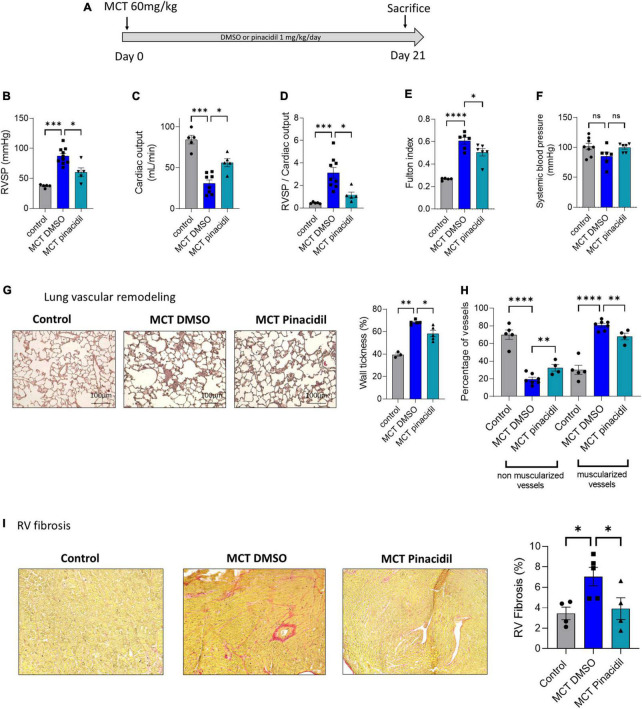
*In vivo* long-term pinacidil treatment interferes with MCT-PH development. **(A)** *In vivo* experimental design. Pinacidil (1 mg/kg/day for 3 weeks) was administered long-term during MCT exposure by intraperitoneal injection. **(B)** RVSP (mm Hg; *n* = 5–10 different rats per condition) **(C)**, Cardiac output (CO; ml/min) (*n* = 5–8 different rats per condition). **(D)** PVR (evaluated by the RVSP/CO ratio) (*n* = 5–8 different rats per condition). **(E)** Fulton index (RV/LV + septum) (*n* = 6 different rats per condition). **(F)** Pulmonary vessel occlusion (%) was analyzed by HES (*n* = 3–5 rats per condition). **(G)** Percentage of non-muscularised and muscularised vessels [100 vessels per rat, (*n* = 4–7 rats)] as measured by immunostaining against α-smooth muscle actin (αSMA) and Von Willebrand Factor (VWF). **(H)** Interstitial fibrosis was analyzed with Sirius red staining in the RV compartment from control, MCT + DMSO, and MCT + pinacidil rats. **(I)** Quantification of the percentage of fibrosis in RV tissue in each condition (*n* = 20 images per rat from 4–5 different rats). ns: non-significant, *P < 0.05, ^**^P < 0.01, ^***^P < 0.001, ^****^P < 0.0001.

**FIGURE 7:**
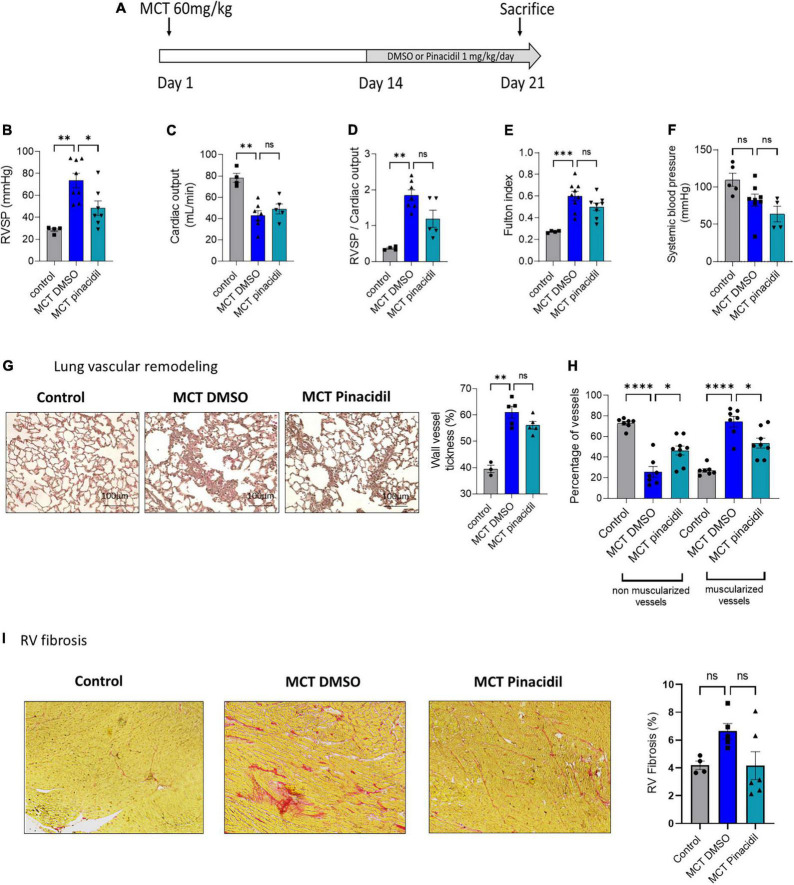
*In vivo* curative pinacidil treatment reduces the development of PH in MCT-PH rats. **(A)** *In vivo* experimental design. Pinacidil (1 mg/kg/day from day 14 to day 21) was administered short-term during MCT exposure by intraperitoneal injection. **(B)** RVSP (mm Hg; *n* = 4–8 different rats per condition) **(C)**, Cardiac output (CO; ml/min) (*n* = 4–7 different rats per condition). **(D)** PVR (evaluated by the RVSP/CO ratio) (*n* = 4–7 different rats per condition). **(E)** Fulton index (RV/LV + septum) (*n* = 4–8 different rats per condition). **(F)** Pulmonary vessel occlusion (%) was analyzed by HES (*n* = 3–5 rats per condition). **(G)** Percentage of non-muscularised and muscularised vessels [100 vessels per rat, (*n* = 6–8 rats)] as measured by immunostaining against αSMA and VWF. **(H)** Interstitial fibrosis was identified with Sirius red staining in the RV compartment from control, MCT + DMSO, and MCT + pinacidil rats. **(I)** Quantification of the percentage of fibrosis in RV tissue in each condition (*n* = 20 images per rat from 4–5 different rats). ns: non-significant, *P < 0.05, ^**^P < 0.01, ^***^P < 0.001, ^****^P < 0.0001.

### 3.6. *In vivo* pharmacological activation of SUR2 increased the heart rate of control rats

To evaluate the consequence of *in vivo* pinacidil exposure on control rats, we exposed chronically healthy rats to 1 mg/kg/d pinacidil for 3 weeks (Figure 5C). *In vivo* chronic exposure to pinacidil had no pulmonary vascular consequences, as indicated by unchanged right ventricular systolic pressure (RVSP), cardiac output (CO), pulmonary vascular resistance (PVR) (evaluated by RVSP/CO ratio), RV hypertrophy (Fulton index), systemic blood pressure (measured in the carotid artery) (Figures 5D–H). Pinacidil administration did not affect pulmonary vessel remodeling and neo-muscularization (Figures 5I, J).

We observed increased heart rate and decreased cycle length of the pinacidil-exposed rats (Table 1) without changes in heart morphometric parameters (Supplementary Table 4), which is in accordance with pinacidil-induced reduced APD in RV cardiomyocytes. PA acceleration time (PAAT) and RV ejection time (RVET) were reduced, while the PAAT/RVET ratio remained unchanged (Table 1). All the other measured parameters were unchanged, including RV and LV thickness and diameters (Table 1).

**TABLE 1:** Evaluation of right ventricular and left ventricular function by echocardiography measured in pinacidil or dimethyl sulfoxide-exposed rats.

	DMSO *n* = 8	Pinacidil *n* = 8
HR (bpm)	326.1 ± 15.42	29 ± 1.2**
Cylce length (ms)	159.4 ± 5.52	143.3 ± 3.19*
RVET (ms)	82.75 ± 2.38	71.63 ± 2.66**
PAAT (ms)	36.00 ± 1.36	29 ± 1.2**
PAAT/RVET	0.44 ± 0.016	0.40 ± 0.007
VTI Pulmonary Artery (ml)	5.30 ± 0.29	4.87 ± 0.16
RV EDd (mm)	6.24 ± 0.26	6.72 ± 0.26
RV EDs (mm)	1.47 ± 0.28	1.84 ± 0.13
RV tihickness (mm)	0.87 ± 0.06	0.93 ± 0.06
RV FS (%)	48.75 ± 4.98	43.95 ± 3.12
VTI Aorta (ml)	7.38 ± 0.28	7.09 ± 0.38
Stroke Volume Aorta (ml)	15.76 ± 1.25	15.17 ± 0.85
LV EDd (mm)	6.24 ± 0.26	6.72 ± 0.26
LV EDs (mm)	3.35 ± 0.22	3.47 ± 0.16
LV thickness (mm)	1.48 ± 0.14	1.50 ± 0.12
LV FS (%)	39.80 ± 4.76	47.70 ± 3.40

HR, Heart rate; RVET, right ventricular ejection time; PAAT, pulmonary artery acceleration time; VTI, velocity-time integral; RV EDd, right ventricular end-diastolic diameter; RV Eds, right ventricular end-systolic diameter; RV FS, right ventricular fractional shortening; LV EDd, left ventricular end-diastolic diameter; LV Eds, left ventricular end-systolic diameter; LV FS, left ventricular fractional shortening. ns, non-significant *P < 0.05, **P < 0.01.

### 3.7. Preventive *in vivo* SUR2 pharmacological activation reduced the development of MCT-induced PH

In this study, SUR2 activation reduced the proliferation rate of PAH-hPASMCs and caused PA relaxation in the MCT-PH rats. Therefore, we assessed the consequences of *in vivo* pharmacological activation of the SUR2 channel in MCT-PH rats (Figure 6A). We administered pinacidil 1 mg/kg/day *in vivo* to MCT-PH rats from days 1 to 21 (Figure 6A). We measured a reduction in the RVSP (Figure 6B), an increase in CO (Figure 6C), a decrease in PVR (Figure 6D), and a reduction in RV hypertrophy in the MCT + pinacidil rats compared to the MCT + DMSO rats (Figure 6E). Further, Dp/dt min was decreased, and heart rate was increased in the MCT + pinacidil rats compared to the MCT + DMSO rats (Supplementary Table 5). At the same time, no difference was observed in systemic blood pressure (Figure 6F).

*In vivo* preventive treatment with pinacidil reduced the thickening of pulmonary wall vessels induced by MCT exposure (Figure 6G) and reduced the neo-muscularisation capacity of pulmonary vessels, as attested by the increased non-muscularised vessels and by the decreased of muscularised vessels (Figure 6H). Preventive pinacidil treatment also reduced RV fibrosis in the MCT + pinacidil rats compared to the MCT-DMSO rats (Figure 6I).

### 3.8. *In vivo* curative pharmacological activation of SUR2 reduced the severity of MCT and chronic-hypoxia (CH)-induced PH

In the curative strategy in the MCT-PH model (day 14 to day 21) (Figure 7A), pinacidil significantly reduced RVSP (Figure 7B), but not CO (Figure 7C), RVSP/CO ratio (Figure 7D), and Fulton index (Figure 7E) not systemic blood pressure (Figure 7F) and other morphometric parameters in the MCT-PH rats (Supplementary Table 6). Additionally, *in vivo* curative treatment with pinacidil reduced the pulmonary vascular remodeling induced by MCT exposure (Figure 7G) and pulmonary vessel neo-muscularisation (Figure 7H). Furthermore, curative pinacidil treatment did not significantly reduce RV fibrosis in the MCT-pinacidil rats compared to the MCT-DMSO rats (Figure 7I). These results suggest that pinacidil treatment may reduce the severity of PH in MCT-exposed rats.

Finally, we analyzed the protein expression of SUR2A, SUR2B, and Kir6.1 in in CH-PH rats. We found that SUR2A, SUR2B, and Kir6.1 lung protein expression were unchanged in CH-exposed rats compared with normoxia rats (Supplementary Figure 4A), like in human lung tissues. Then, we evaluated the consequences of *in vivo* pharmacological activation of the SUR2 by pinacidil in CH-PH rats (Supplementary Figure 4B). We treated with 1 mg/kg/day of pinacidil CH-PH rats from days 14 to 21. We measured an improvement in the RVSP (Supplementary Figure 4C), CO (Supplementary Figure 4D), and PVR (Supplementary Figure 4E), but not Fulton index, systemic blood pressure, and Heart rate (Supplementary Figures 4F–H). The pulmonary vessel wall thickness and the pulmonary vessel neomuscularization were reduced by the pinacidil treatment (Supplementary Figures 4I, J).

## 4. Discussion

In this study, we report on several essential findings related to SUR2/Kir6.1 expression and function in the PAH pathogenesis. First, SUR2A, SUR2B, and Kir6.1 were expressed in hPASMCs and hPAECs. Second, SUR2 activation reduced the proliferation of control-hPASMCs, control-hPAECs, and PAH-hPASMCs and the migration of control-hPASMCsc. Third, pharmacological activation of SUR2 produced PA relaxation in both the control and PH rat models. Fourth, SUR2A and Kir6.1 were highly expressed at the RV level and contributed to potential repolarization in the control rats. Fifth, chronic *in vivo* SUR2 activation with pinacidil increased heart rate without disturbing pulmonary circulation hemodynamics in the control rats. Finally, *in vivo* activation of SUR2 reduced the development of PH in two preclinical models of PH (MCT- and CH-PH rat models).

### 4.1. SUR2A/SUR2B localization expression

SUR2A shares 99% of homology with SUR2B (25). However, they have different localization and sensitivities to ATP. In the absence of pinacidil, when SUR2B is associated with Kir6.1, this channel is stimulated by ADP and ATP rather than inhibited by ATP (26). Moreover, the half-maximal inhibitory concentration of ATP for SUR2A and SUR2B are 100 and 300 μmol/L, respectively (27). Regarding their different localization, SUR2A is the primary isoform in the heart, skeletal muscle, and brain (28, 29), while SUR2B is more ubiquitous. The *in situ* hybridization technique indicated that SUR2B and Kir6.1 mRNA are found in many systemic conditions but not in PAs (30). Here, we demonstrated for the first time that SUR2A, SUR2B, and Kir6.1 are expressed at the protein level in human and rat lung tissues and isolated hPAECs and hPASMCs from controls and patients with PAH. SUR2A and SUR2B are predominantly associated with Kir6.1, but they can also be associated with Kir6.2, commonly associated with SUR1. We recently found that Kir6.2 is expressed in control and PAH hPAECs and hPASMCs and that Kir6.2 is increased in PAH-hPASMCs and lungs of MCT-PH rats (8). In the pulmonary vasculature, we could reasonably imagine that SUR2A or SUR2B can co-assemble with Kir6.1 and Kir6.2. Therefore the increase in Kir6.2 observed in the context of PAH may modify the properties of KATP (8).

Moreover, as suggested by Videbaek et al. (31) pinacidil is more potent in systemic and rat pulmonary arteries. In the present study, we confirmed that pinacidil application is more potent to relax the aorta artery than the pulmonary artery. KATP channels, including SUR2A and SUR2B, are expressed in the mitochondrial membrane (32) and may inhibit the mitochondrial radical oxygen species (ROS) production (33). As ROS are overproduced in PAH (34), we could hypothesize that SUR2 pharmacological activation reduces the severity of PH partly due to an inhibition of ROS production.

### 4.2. SUR2 function in the RV

SUR2, Kir6.1, and Kir6.2 are found in the heart (35). However, all current studies on the role of SUR2 in the heart involve the LV compartment. We found that RV cardiomyocytes also expressed SUR2A, Kir6.2, and Kir6.1 (8), while SUR2B expression appeared low in the RV compared to the LV. LV cardiomyocytes from guinea pigs administered with pinacidil showed significantly shortened APD (36). Inversely, LV cardiomyocytes isolated from *kir6.2^–/–^* mice displayed a prolonged APD in high glucose concentration compared with cardiomyocytes from Wild-Type mice (37).

Moreover, in the LV of control rats, SUR2A and SUR2B appear localized in the mitochondria, indicating that KATP could also exert cardio-protection based on their role as KATP in the mitochondria (32). The role of the SUR2A/Kir6.1 channel in APD and the mitochondrial function of cardiomyocytes may have cardioprotective consequences that could benefit from RV dysfunction occurring in MCT-PH models (18, 20). Indeed, Storey et al. demonstrated that KATP was crucial for regulating cardiomyocyte Ca^2+^ homeostasis under basal and pathological conditions. The reduction of KATP current increases Ca^2+^ overload by preventing mitochondrial membrane potential oscillations during oxidative stress (38). Here, we demonstrated that SUR2 activation leads to a reduction of APD in control RV cardiomyocytes. As we previously found, a pathological prolongation of APD in RV cardiomyocytes isolated from MCT-PH rats (39), we could hypothesize that the protective effect of SUR2 activation in the MCT-PH model could partly act by these mechanisms in RV cardiomyocytes. In LV cardiomyocytes, SUR2 activation by pinacidil was already demonstrated to modulate the action potential duration (40, 41). Additionally, we found that chronic pinacidil application in healthy rats enhances the heart rate of pinacidil-exposed rats, suggesting a potential direct action in the sinoatrial node, which is the dominant pacemaker in the mammalian heart. It was previously demonstrated that Kir6.1 containing KATP channels contributes to the sinoatrial node excitability and heart rate control (42).

### 4.3. *ABCC9* in Cantu syndrome

Recently, the gain-of-function mutations in *ABCC9* is the most important genetic cause of Cantu syndrome, characterized by congenital hypertrichosis, osteochondroplasia, cardiomegaly, dilated vasculature, and pericardial effusion. Interestingly, some patients with Cantu syndrome also develop pulmonary hypertension (43–46). Twelve different *ABCC9* mutations are gain-of-function (43) or LOF-of-function mutations (47). The pathophysiological mechanism involving the development of PH is complex, suggesting that the constitutive SUR2A/Kir6.1 opening leads to systemic vasorelaxation and hypotension, leading to compensatory cardiac hypertrophy and hypercontractility and PH (35). Moreover, some patients have also been diagnosed with PAH due to LV disease (48). This study found that daily pinacidil administration in control rats at 1 mg/kg/day for 3 weeks had no consequences on hemodynamic parameters and pulmonary vascular remodeling. Previous work suggested that long-term treatment (preventive protocol) with KATP openers treatment reduced the severity of PH (12–14). Our results demonstrated that curative SUR2 activation could be an interesting therapy for PAH.

## 5. Conclusion

We showed that pharmacological activation of SUR2 reduces the proliferation and migration capacity of PAH-hPASMCs and that SUR2 contributes to PA tone. Moreover, *in vivo* SUR2 curative activation effectively restores or ameliorates PH in MCT-PH and CH-PH rat models. Despite the SUR2 gain-of-function mutation being a cause of cardiovascular abnormalities observed in patients with Cantu syndrome, our results showed that pharmacological activation of SUR2 should be considered for treating PAH.

## 6. Limitations

Since SUR2 is expressed by other cell types such as fibroblasts or other tissues (liver, for example (28, 49), our results did not exclude that *in vivo* SUR2 activation reduces PH by acting in pulmonary and cardiac fibroblasts (50) or by improving liver function in MCT-PH model, which is characterized by liver dysfunction. The generation of cardiac or smooth muscle cell SUR2 overexpressing animals will constitute a powerful tool. As indicated in the Supplementary Table 1, our samples (lung, PAECs, PASMCs) used in the study were from control and PAH patients with different ages, so we did not exclude that these differences have any consequences on SUR2/Kir6.1 expression. However, using a human sample, there is no alternative strategy.

## Data availability statement

The original contributions presented in this study are included in the article/Supplementary material, further inquiries can be directed to the corresponding author.

## Ethics statement

The studies involving human participants were reviewed and approved by Protocol N8CO-08- 003, ID RCB: 2008-A00485-50, approved on June 18, 2008. The patients/participants provided their written informed consent to participate in this study. The animal facility is licensed by the French Ministry of Agriculture (agreement No. C92-019-01).

## Author contributions

HL, VC, DM, and FA performed conception and design. HL, BM, MD, ABo, ABe, JS, VD, VC, and FA conducted the experiments and performed the data analysis. All authors drafted and brought the important intellectual content of the manuscript and reviewed and approved the final version of the manuscript.
