# Supplementary material for: Involvement of SUR2/Kir6.1 channel in the physiopathology of pulmonary arterial hypertension
Source: Front Cardiovasc Med. 2023 Jan 10;9:1066047. doi: 10.3389/fcvm.2022.1066047 (PMC9871631; doi:10.3389/fcvm.2022.1066047)
Supplement: Supplementary file 1 [file Data_Sheet_1.docx]

**Involvement of SUR2/Kir6.1 channel in the physiopathology of pulmonary arterial hypertension**

Short title: SUR2/Kir6.1 in PAH

Hélène Le Ribeuz^1,2^, Bastien Masson^1,2^, Mary Dutheil^1,2,3^, Angèle Boët^1,2^, Antoine Beauvais^1,2^, Jessica Sabourin^4^, Vincent Thomas De Montpreville^5^ Véronique Capuano^1,2,3^, Olaf Mercier^6^, Marc Humbert^1,2,7^ David Montani^1,2,7^ and Fabrice Antigny^1,2^

*^1^Université Paris-Saclay, Faculté de Médecine, Le Kremlin-Bicêtre, France (H.L-R, M.D, A.B, V.C, M.H, D.M, and F.A)*

*^2^INSERM UMR_S 999 « Hypertension pulmonaire : Physiopathologie et Innovation Thérapeutique », Hôpital Marie Lannelongue, Le Plessis-Robinson, France (H.L-R, M.D, A.B, V.C, M.H, D.M, and F.A)*

*^3^Hôptal Marie Lannelongue, Groupe Hospitalier Paris Saint-Joseph, Le Plessis Robinson, France (M.D, V.C)*

*^4^Inserm, UMR-S 1180, Signalisation et Physiopathologie Cardiovasculaire, Université Paris-Saclay, Châtenay-Malabry, France (J.S)*

*^5^Department of Pathology, Groupe Hospitalier-Marie Lannelongue, 92350 Le Plessis-Robinson, France (V.D-M)*

*^6^Service de Chirurgie Thoracique, Vasculaire et Transplantation Cardio-Pulmonaire, Hôpital Marie Lannelongue, Groupe Hospitalier Paris Saint Joseph, Le Plessis Robinson, France. (O.M)*

*^7^Assistance Publique - Hôpitaux de Paris (AP-HP), Service de Pneumologie et Soins Intensifs Respiratoires, Centre de Référence de l’Hypertension Pulmonaire, Hôpital Bicêtre, Le Kremlin-Bicêtre, France. (M.H, D.M)*

Corresponding author: Fabrice Antigny, INSERM UMR_S 999, Hôpital Marie Lannelongue, 133, Avenue de la Résistance, F-92350 Le Plessis Robinson, France. Tel.: (33) 1 40 94 22 99, e-mail: [fabrice.antigny@inserm.fr](mailto:fabrice.antigny@inserm.fr)

**Supplemental tables**

Supplemental table 1: Characteristics of Controls and PAH patients before lung transplantation

|  | **PAH (n=12)** | **Control (n=10)** |
| --- | --- | --- |
| **Age (years)** | **22 [45] 62** | **49 [65] 83** |
| **Sex (Males vs Females)** | **2M /10F** | **3M / 7F** |
| **Mutation in *BMPR2* gene** |  |  |
| **carrier** | **1** | **NA** |
| **no-carrier** | **11** | **NA** |
| **mPAP (mmHg)** | **41 [59.83] 85** | **NA** |
| **CI (l/min/m^2^)** | **2.23 [3.14] 6** | **NA** |
| **PVR (Wood units)** | **8.4 [12.86] 36** | **NA** |
| **PCP (mmHg)** | **6 [9.08] 14** | **NA** |
| **Medications:** |  |  |
| **tritherapies** | **80%** | **NA** |
| **bitherapies** | **5%** | **NA** |
| **monotherapies** | **0%** | **NA** |

Supplement Table 2: Antibodies used for western blotting and Immunofluorescence

| Antibody | Species | Dilution WB | Dilution IF | Supplier | Reference |
| --- | --- | --- | --- | --- | --- |
| Kir6.1 | Rabbit | 1/1000 | 1/100 | Alomone labs | APC-105 |
| SUR2A | Rabbit | 1/1000 | 1/100 | Novus biologicals | NBP1-59350 |
| SUR2B | Mouse | 1/1000 | x | Neuromab | Q63563-2) |
| SUR2B | Mouse | x | 1/100 | sigma | MABN511 |
| β-actin-HRP | Mouse | 1/2000 | x | Santa cruz | sc-47778 |
| α-SMA-FITC | Mouse | x | 1/200 | sigma | A2547 |
| VWF | Rabbit | x | 1/200 | Dako | A0082 |

Supplemental Table 3: Primers used for RT-qPCR

| Gene | Primer reference (Applied Biosystem) | Species |
| --- | --- | --- |
| 18S | [Hs03003631_g1](https://www.thermofisher.com/taqman-gene-expression/product/Hs03003631_g1?CID=&ICID=&subtype=) | Human |
| ABCC9 | [Hs00245832_m1](https://www.thermofisher.com/taqman-gene-expression/product/Hs00245832_m1?CID=&ICID=&subtype=) | Human |
| ABCC9 | [Rn00564842_m1](https://www.thermofisher.com/taqman-gene-expression/product/Rn00564842_m1?CID=&ICID=&subtype=) | Rat |
| KCNJ8 | [Hs00958961_m1](https://www.thermofisher.com/taqman-gene-expression/product/Hs00958961_m1?CID=&ICID=&subtype=) | Human |
| KCNJ8 | [Rn01492857_m1](https://www.thermofisher.com/taqman-gene-expression/product/Rn01492857_m1?CID=&ICID=&subtype=) | Rat |

Supplemental Table 4: Morphometric and functional parameters of heart in DMSO and pinacidil exposed rats

Supplemental Table 5: Morphometric and functional parameters of heart in control, MCT + DMSO and MCT + pinacidil rats (long term of treatment)

Supplemental Table 6: Morphometric and functional parameters of heart in control, MCT + DMSO and MCT + pinacidil rats (short term of treatment)

**Supplemental figure legends**

**
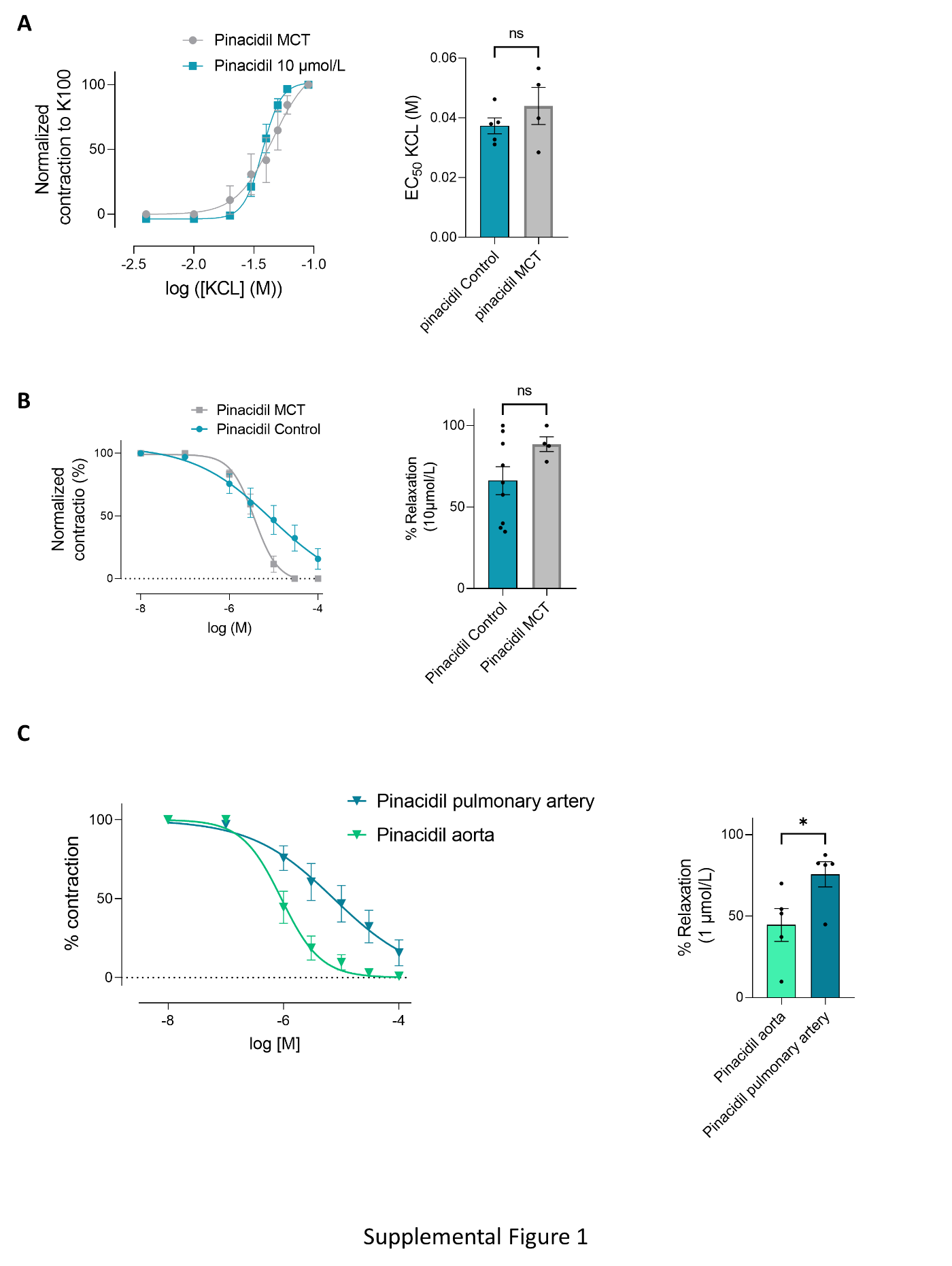
**

**Supplemental Figure 1**: **Consequence of pinacidil application on pulmonary arterial tone.** (A) Dose-response curve (normalized to K100) was established by applying increasing concentrations of potassium chloride (KCl) to isolated PAs from control and MCT rats in the presence of pinacidil at 10 µmol/L) Corresponding quantification of Hillslope and EC50 values (n=4-5 rats). (B) Dose-response to pinacidil (100 nmol/L to 100µmol/L) on precontracted control and MCT rat PA by 1 µmol/L of U46619. Graphics represent the contraction percentage at 10 µmol/L of PA of control or MCT rats treated with pinacidil (n =5-9 rats). (C) Dose-response to pinacidil (100 nmol/L to 100µmol/L) on precontracted control rat aorta by 1 µmol/L of U46619. Graphics represent the contraction percentage at 10 µmol/L of the Aorta and PA of control rats treated with pinacidil (n =5 different rats). ns: non-significant.

**
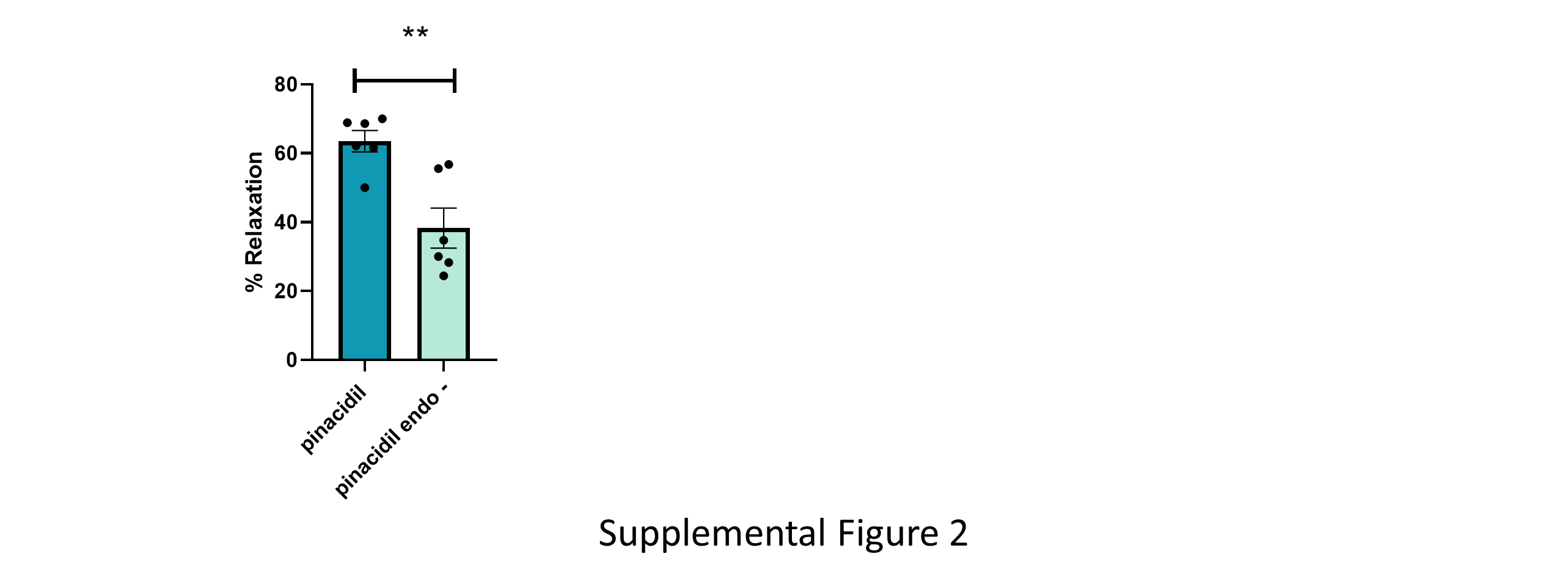
**

**Supplemental Figure 2**: **Pinacidil mediated pulmonary artery relaxation in the absence of pulmonary endothelium** Quantification of pinacidil mediated PA relaxation with (endo +) or without pulmonary endothelium (endo -) (n=7 different rats). ns: non-significant. ***P*<0.01.

**
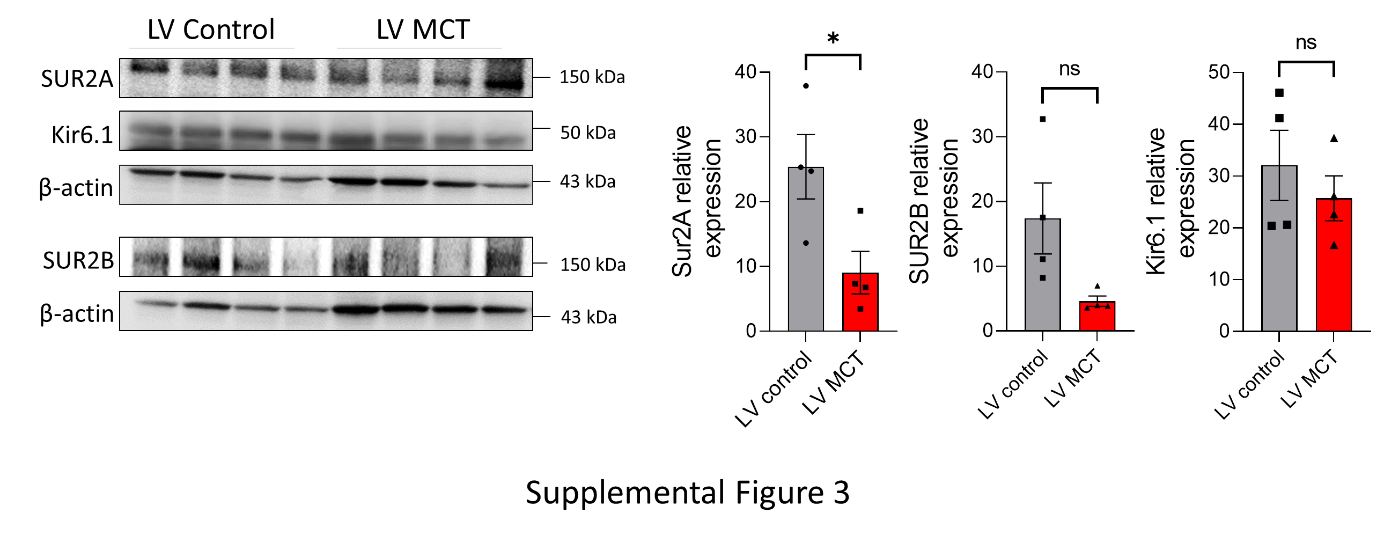
**

**Supplemental Figure 3: SUR2A, SUR2B, and Kir6.1 protein expression in RV and LV compartments.** Representative Western blots of SUR2A, SUR2B, and Kir6.1 in LV from control and MCT-PH rats (3 weeks), and quantification of SUR2A, SUR2B, and Kir6.1 in LV from control and MCT-PH rats (n=4-). ns = non-significant; **P*< 0.05 control.

**
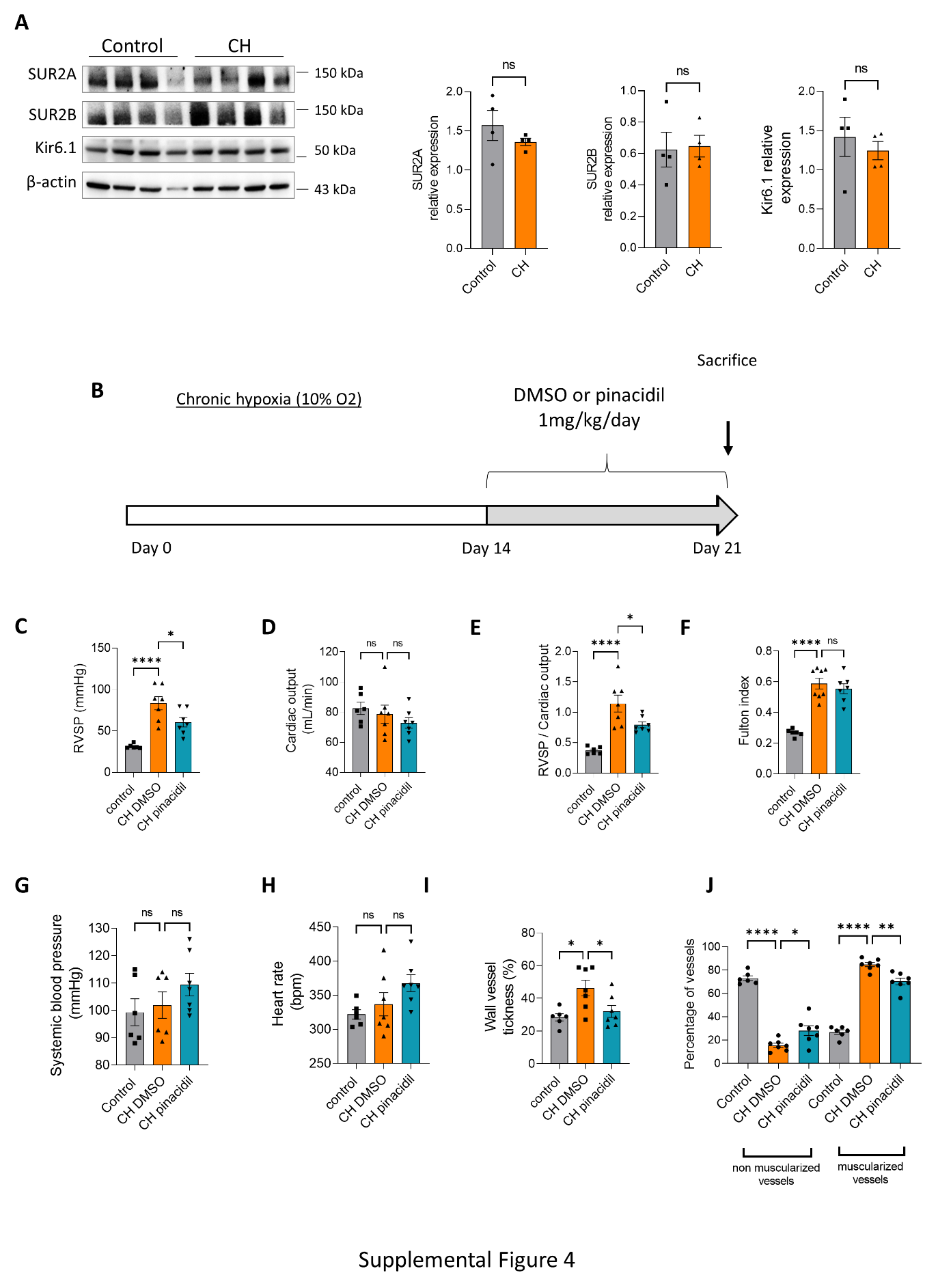
**

**Supplemental Figure 4: *In vivo* curative pinacidil treatment reduces the development of PH in Chronic-Hypoxia (CH)-PH rats***.* (A) Representative western blots and quantification of SUR2A, SUR2B, and Kir6.1 in lungs from control and CH-PH rats (3 weeks) (n=4). (B) *In vivo* experimental design. Pinacidil (1 mg/kg/day from day 14 to day 21) was administered short-term during CH exposure by intraperitoneal injection. (B) RVSP (mm Hg; n = 6–7 different rats per condition) (C), Cardiac output (CO; mL/min) (n = 6–7 different rats per condition). (D) PVR (evaluated by the RVSP/CO ratio) (n = 6–7 different rats per condition). (E) Fulton index (RV/LV+septum) (n = 6–7 different rats per condition). (F) Systemic blood pressure (measured in carotid artery mm Hg; n = 6-7 different rats per condition) (G) Heart rate (bpm; n = 6-7 different rats per condition) (H) Pulmonary vessel occlusion (%) was analysed by HES (n = 6–7 rats per condition). (I) Percentage of non-muscularised and muscularised vessels (100 vessels per rat, (n = 6–7 rats) as measured by immunostaining against αSMA and VWF. ns: non-significant, *P<0.05 ***P*<0.01****P*<0.001, *****P*<0.0001.
